# Enhanced Memory for Fair-Related Faces and the Role of Trait Anxiety

**DOI:** 10.3389/fpsyg.2019.00760

**Published:** 2019-04-16

**Authors:** Gewnhi Park, Benjamin U. Marsh, Elisha J. Johnson

**Affiliations:** Department of Psychology, Azusa Pacific University, Azusa, CA, United States

**Keywords:** fair-modulated memory, economic decision task, trait anxiety, the ultimatum game, anxiety-modulated fair memory

## Abstract

The current research examined whether fair consideration—a social norm that people inherently prefer to confirm—would modulate face recognition. Each neutral face was associated with fair or unfair offers via an economic decision task, the Ultimatum Game (UG) task. After the UG, participants were asked to identify the faces of proposers who made different offers. Enhanced memory was observed for fair-related compared to unfair-related faces. Furthermore, high trait anxiety was associated with reduced memory for fair-related faces. These results were further confirmed by signal detection theory. The current research provided initial evidence that people showed enhanced memory for faces that made fair offers from the economic decision task, and that high trait anxiety was associated with reduced fair-related memory. Possible neural mechanisms and the implication in economic and social situations have been discussed.

## Introduction

Human faces provide important social and biological information critical for shaping social behavior (Park et al., 2012; Wiese et al., 2013). People demonstrate a remarkable ability to discriminate and recognize human faces, which allows for successfully navigating the social world (Park et al., 2012; Wiese et al., 2013). Also, people are capable of attuning their ability to detect particular information about faces through experiences (Zebrowitz and Montepare, 2006). The current research examined whether fair consideration—a social norm that people inherently prefer to confirm—would modulate face recognition (Gächter et al., 2017). Furthermore, there is growing evidence that trait anxiety plays a role in socioeconomic decision making that relies on the social norm of fairness (Grecucci et al., 2013). In the current experiment, we examined whether trait anxiety would modulate the impact of fair consideration on subsequent face recognition.

### Theoretical Accounts of Face Perception

To understand the cognitive and neural mechanisms of face recognition, people initially relied on the dual process model (Bruce and Young, 1986). The dual process model suggested that there are two distinctive functional routes for the recognition of invariant facial identity and variant facial expressions (Bruce and Young, 1986). Neuroimaging evidence has identified the inferior occipital gyri and superior temporal sulcus (STS) as neural mechanisms for analyzing the facial expression and the inferior occipital gyri and lateral fusiform gyrus for facial identity (Haxby et al., 2000). As such, the dual process model obtained converging evidence from the neuroimaging studies and has been widely accepted (Calder and Young, 2005).

However, the need for a more comprehensive model of face recognition in the social context called for incorporating Gibsonian theories of perception (Gibson, 1966, 1979; Zebrowitz, 2006). The ecological theory emphasized the function and utility of human perception on guiding behavior in social contexts and highlighted the role that face recognition plays in guiding adaptive social behavior and achieving goals (McArthur and Baron, 1983; Zebrowitz, 2006). For example, people demonstrated a remarkable ability to detect various social attributes from physical appearance, including deceptive intent (Runeson and Frykholm, 1983), motivational incentives (Schultheiss et al., 2005), motion (Johansson, 1973), personality, social relationships, sexual orientation, and teaching effectiveness (Johansson, 1973; Ambady et al., 2000). This ability to detect these attributes would help to determine how to behave in various social contexts and guide social interaction. Not only that but also people developed the ability to recognize particular psychological traits through experiences, which would help to guide interpersonal interaction and navigate the social world (Zebrowitz, 2006). In this experiment, we examined whether and how previous experiences of fairness would shape face recognition.

### Fair Consideration and the Ultimatum Game

People inherently prefer the fair distribution of resources and are antagonized by unfair distributions (Fehr and Schmidt, 1999; Fehr and Camerer, 2007; Güroğlu et al., 2011). Moreover, the assessment of fairness shapes and guides future social interaction and interpersonal relationships: People prefer those who treat them fairly and are angry at those who treat them unfairly (Güroğlu et al., 2011). According to the norm compliance framework, fair consideration is an important social norm that people show a robust preference, even at the expense of monetary sacrifice (Gächter et al., 2017). For example, in an economic decision game known as the Ultimatum Game (UG) task, people are willing to lose money for the sake of promoting fairness. In the UG task, the proposer is given a sum of money, $10, and makes offers to the responder as to how to split the money between themselves (Sanfey et al., 2006; Scheres and Sanfey, 2006). Some offers are fair so that the money is evenly split between the proposer and the responder ($5:$5). However, other offers are unfair, such that the proposer receives more money and the responder receives less money ($9:$1, $8:$2, or $7:$3). Then, the responder decides whether to accept or reject the offer. When the responder accepts the offer, the money will be split between the two players according to the offer. When the responder rejects the offer, neither receives anything. Therefore, the rational response for the responder is to accept any offer because any monetary reward is preferable to none. However, extensive research has shown that the responders frequently rejected unfair offers ($9:$1, $8:$2, or $7:$3), even if they would not receive anything (Pillutla and Murnighan, 1996; Sanfey, 2003; Tabibnia et al., 2008). The rejection of unfair offers was generally construed as “altruistic punishment” for norm violation (Fehr and Rockenbach, 2004; Frith and Singer, 2008). The responders experienced an unpleasant emotion in response to unfair offers and were willing to punish the proposers who made unfair offers by depriving them of getting a greater share of the money, even at the risk of forfeiting monetary profit (Fehr and Rockenbach, 2004; Frith and Singer, 2008).

### Overview

Previous research has shown that fair consideration plays an important role in economic decision-making. However, relatively little is known about whether and how fairness associated with the economic game would influence face recognition. Fair-related faces carry important social and normative value. Moreover, the assessment of fairness shapes and guides future social interaction and interpersonal relationships (Güroğlu et al., 2011). According to the ecological theory (Zebrowitz, 2006), face recognition serves adaptive function and utility that help people to navigate the social world and achieve goals (McArthur and Baron, 1983; Zebrowitz, 2006). Fair-related faces are inherently favored, socially normative, and highly functional in guiding social behavior (Güroğlu et al., 2011). For example, recognizing people who make fair offers are advantageous for future economic dealings because they are more likely to make fair business deals in the future. Furthermore, it is more likely that people who promote fairness abide by high ethical and moral principles, which helps to build a meaningful interpersonal relationship. Because of its high utility and function in the social world, we predicted that people would be more likely to remember people who make fair offers in a memory task subsequently following the UG task.

Furthermore, we were interested in whether individual differences in trait anxiety would influence fairness-modulated face recognition. There is emerging evidence showing that anxiety modulated the acceptance rate of offers in the UG task, although the results are conflicting (Grecucci et al., 2013; Luo et al., 2014). There is evidence that individuals clinically diagnosed with anxiety disorders were more likely to accept unfair offers compared to individuals without anxiety disorders (Grecucci et al., 2013; Wu et al., 2013). The authors attributed this result to the reluctance to face social confrontation to reinforce the social norm of fairness (Grecucci et al., 2013). Individuals with an anxiety disorder are more likely to experience negative arousals while confronting fairness norm violation. As a result, they are reluctant to confront and to punish those who violated the fairness norm, thus being more likely to accept unfair offers. Individuals with anxiety disorders were more likely to accept unfair offers made by human partners while being more likely to reject unfair offers made by computer partners (Wu et al., 2013). However, this pattern was less obvious in participants with sub-clinical anxiety. Luo et al. (2014) reported that high-anxious individuals were more likely to reject unequal offers made by human counterparts compared to computer counterparts. However, no report was made about the difference in acceptance rates between fair and unfair offers.

We speculated that anxiety would modulate the impact of fair consideration on face recognition. It has been well documented that individuals with high trait anxiety demonstrated different cognitive bias, including memory bias, favoring threatening stimuli (Clark, 1999; Wilson et al., 2006; Bishop, 2009; Van Bockstaele et al., 2014). Individuals with high anxiety would want to minimize the advantage and importance of fairness to avoid anxiety associated with confronting and punishing fair norm violators. As a result, individuals with high anxiety may show reduced memory for the proposers who made fair offers.

## Materials and Methods

### Participants

One hundred ten undergraduate students (74 women; mean age = 19.9, *SD* = 1.66) successfully completed the experiment for partial course credit^1^. People with a history of emotional disorders (e.g., anxiety and depression) were excluded from this experiment. We excluded the data of one participant who had very poor recognition accuracy (less than 20%), resulting in 109 participants. The ethics committee approved the study.

### Procedure

#### The Ultimatum Game

**FIGURE 1:**
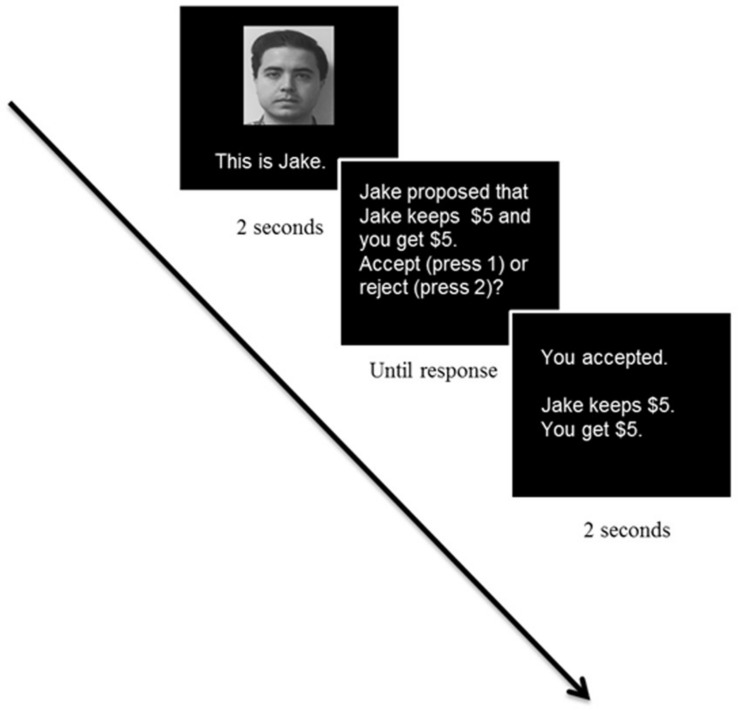
Sample trial in the Ultimatum Game Task (A written consent has been obtained from the individual for the publication of the image).

Participants played a modified version of the ultimatum game (UG) which was adopted from van’t Wout and Sanfey (2011). The UG was programmed in E-prime software (Psychology Software Tools, Pittsburgh). Before starting the experiment, participants were given the rules of the ultimatum game, read the instructions and completed three practice trials to ensure the participants fully understood the game. On each trial, participants were first presented with a picture of their human opponent. After the proposal was presented, participants could respond by a button and chose to press accept or reject (the offer). There was a total of 24 trials that participants played a role as a responder (see Figure 1 for an example of a full trial). Twenty-four trials consisted of 6 fair offers ($5 to each player) and 18 unfair offers defined as offering the participant less than half of the money. The unfair set consisted of six offers of $3, six offers of $2, and six offers of $1. We did not include $4 offers because $4 offers are generally perceived as fair and thus frequently accepted. Forty-eight neutral male faces were selected from the NimStim Face Stimulus Set (MacArthur Foundation Research Network on Early Experience and Brain Development). The offers were made by male partners to avoid the gender effect (Kim et al., 2012), and the order of partners and the pictures associated with each offer was randomized. Participants were not informed of the total number of trials in advance. The instructions emphasized that different partners in the game would play the game independently of each other, and participants were told that the games would be played with the set of partners they saw. To encourage participants to make decisions seriously, participants were told that they would be paid 5% of the total amount of money earned in the game in addition to course credit. After the UG task, participants completed the filler task to create time delay before the surprise recognition task.

#### Filler Task

For the filler task, participants completed several personality questionnaires, including the Duke University Religion Index (Koenig and Büssing, 2010), the Difficulties in Emotion Regulation Scale (Gratz and Roemer, 2004), and the behavioral inhibition system (BIS) and the behavioral activation or behavioral approach system (BAS) scale (Carver and White, 1994). The filler task took 5 to 10 min^2^.

#### The Surprise Recognition Task

On the completion of the filler task, participants performed the surprise face recognition task in which participants were asked to identify the faces that made offers during the ultimatum task. Participants saw 48 faces (24 old faces and 24 new faces, which were taken from the NimStim Face Stimulus Set) and were asked to indicate with a button press whether each face was one of the faces they saw during the UG task (*Yes* or *No*). Faces were presented in random order and participants proceeded the recognition test at their own pace. Response accuracy and latency were recorded. We report the estimated proportion of accurate responses such that higher scores reflect greater accuracy (1.0 = perfect accuracy). After the task, participants completed the Spielberger State-Trait Anxiety Inventory (Spielberger et al., 1983).

#### The Spielberger State-Trait Anxiety Inventory

The State–Trait Anxiety Inventory (STAI) is a widely used self-report scale to measure anxiety. The STAI state focuses on how respondents feel “right now, at this moment” (e.g., “I feel at ease”; “I feel upset”), and the STAI Trait targets how respondents “generally feel” (e.g., “I am a steady person”; “I lack self-confidence”; Spielberger, 1989). The STAI consists of two 20-item, each of which can be rated on the basis of a 4-point Likert scale, ranging from not at all to very much so for the STAI State and from almost never to almost always for the STAI Trait (Spielberger, 1989). The STAI has excellent internal consistency (average ααs > 0.89), and especially, the STAI Trait has strong test-retest reliability (average *r* = 0.88) at multiple time intervals (Barnes et al., 2002).

## Results

### The Ultimatum Game

**FIGURE 2:**
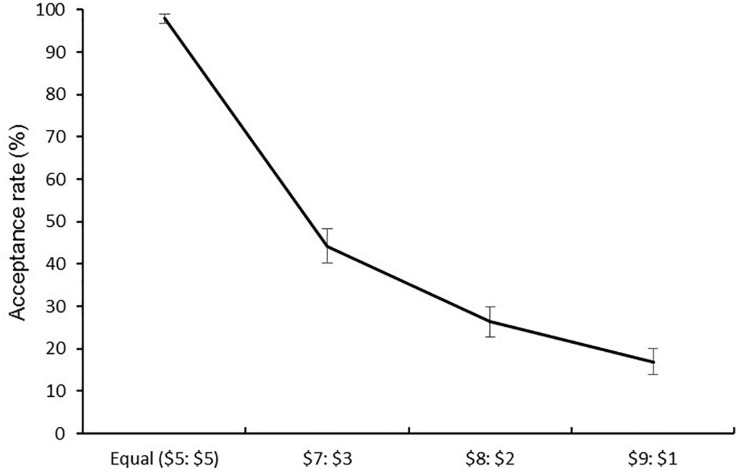
Percentage of acceptance of the different offers.

Replicating previous research (van’t Wout et al., 2010), fair offers ($5) were almost always accepted, $5-$5: *M* = 97.9% (*SD* = 11.1), and acceptance rates decreased as the offers became progressively more unfair: $7-$3: *M* = 44.9% (*SD* = 43.1); $8-$2: *M* = 26.6% (*SD* = 38.2); $9-$1: *M* = 17.1% (*SD* = 32.2; see Figure 2). To assess the relationship between individual differences in trait anxiety and the acceptance rates in the UG, we conducted a 4 (Offers: $5-$5, $7-$3, $8-$2, $9-$1) repeated measures analysis of covariance (ANCOVA) with z-standardized trait anxiety (STAI-trait) as a covariate^3^. There was no interaction between trait anxiety and type of offers (*p* = 0.60). Contrary to previous research linked high anxiety to more acceptance of the unfair offers, we did not find the relationship between anxiety and responses to unfair offers.

### The Surprise Recognition Task

We analyzed the hit rates of faces who made different offers ($5-$5, $7-$3, $8-$2, $9-$1) and new faces, (i.e., correct rejections) using Friedman’s test, a non-parametric alternative to analysis of variance (ANOVA; see Table 1). There was a significant difference among the four conditions, *χ*^2^(3) = 124.65, *p* < 0.001. *Post hoc* analysis with Wilcoxon matched-pairs signed-rank tests were conducted with a Bonferroni correction applied. Participants recognized significantly more faces that made $5-$5 (*Mdn* = 0.83) than faces that made $9-$1 (*Mdn* = 0.50), *Z* = 7.21, *p* < 0.001, *r* = 0.69, and $8-$2 (*Mdn* = 0.67), *Z* = 6.99, *p* < 0.001, *r* = 0.67. However, there was no difference between $5-$5 and $7-$3 (*Mdn* = 0.83; *p* = 0.27). Thus, as predicted, people demonstrated superior memory for faces that made fair offers ($5-$5) than those that made unfair offers ($9-$1 and $8-$2).

**TABLE 1:** Mean accuracy as a function of different types of faces during the recognition memory task.

		**M**	***SD***
New faces		0.90	0.09
Old faces	Fair-related ($5:$5)	0.84	0.19
	Unfair-related (Total)	0.67	0.16
	$7:3	0.82	0.15
	$8:2	0.60	0.23
	$9:1	0.58	0.24

**FIGURE 3:**
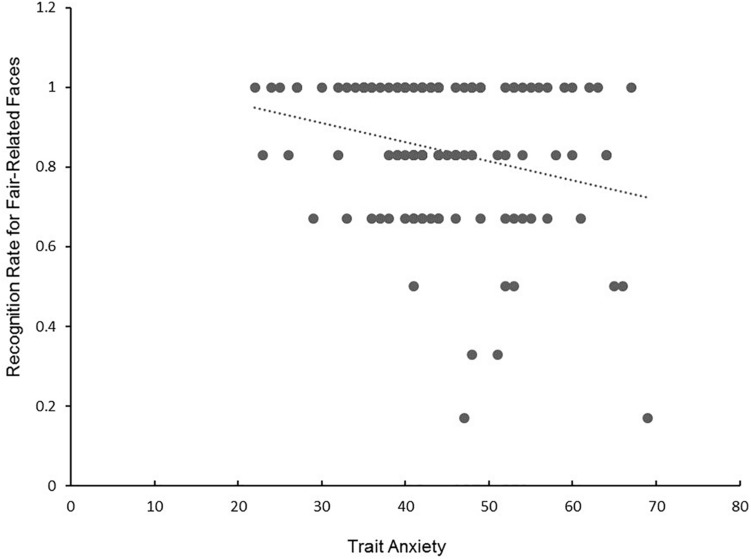
A scatterplot indicating a negative correlation between trait version of the STAI (STAI-trait) (*x*-axis) and recognition rates for fair-related faces (y-axis). *r* = –0.25, *p* < 0.01.

To assess whether individual differences in trait anxiety modulated subsequent fair-related memory, we conducted the Quade test, one of the most frequently cited non-parametric alternative to repeated measures analysis of covariance (Quade, 1967; Rheinheimer and Penfield, 2001), on the hit rates of three types of faces (fair-related, unfair-related, new faces) with trait anxiety (STAI-trait) as a covariate. There was the significant two-way interaction between type of faces and trait anxiety, *F*(2, 324) = 72.63, *p* < 0.001. When trait anxiety was correlated with the hit rates of different types of faces, trait anxiety was more negatively correlated with accuracy for fair-related faces (*r* = -0.25, *p* < 0.01; see Figure 3), but neither with new faces (*r* = 0.01, *p* = 0.94) nor with unfair-related faces (r = -0.06, *p* = 0.58). ^4^ Therefore, consistent with our predictions, participants with high trait anxiety had difficulty recognizing fair-related faces.

#### Signal Detection Theory

Additional analyses using Signal Detection Theory were conducted to further examine the relationship between trait anxiety and recognition memories of fair and unfair faces. Signal Detection Theory decomposes a person’s performance into *discrimination accuracy* (*d’*) and *response criteria* (McNicol, 1972; Macmillan and Creelman, 1991). *d’* refers to how well a person is capable of correctly recognizing a target and rejecting distractors, (i.e., new faces that were not associated with an offer), which reflects the processing of pure memory (Miller and Lewis, 1977). A larger *d’* indicates superior recognition of a target. Response criterion (*C*) reflects the tendency to respond to whether a stimulus has been presented before or not. A positive response criterion indicates a conservative response criterion which is biased toward judging any stimulus as not being presented before (new), thereby producing fewer hits and fewer false alarms. In contrast, a negative response criterion indicates that a lax criterion is adopted, which is biased toward judging any stimuli as being presented before (old), thereby producing more hits and more false alarms. As such, Signal Detection Theory allowed us to tease apart memory processing and decision criterion and to investigate the specific components involved in the process of fair, unfair, and new faces (Miller and Lewis, 1977).

Following Macmillan and Creelman (1991), we computed *d’*, and response criterion (*C*; see Table 2)^5^. For *d’*, there was a significant main effect of the type of faces, *F*(2, 206) = 136.38, *p* < 0.001, *η_p_*^2^ = 0.57. Paired *t*-tests with Bonferroni correction (2-tailed) revealed that participants showed higher *d’* to fair-related (*M* = 2.95, *SD* = 0.86) compared to unfair-related faces (*M* = 1.33, *SD* = 0.53), *t*(108) = 18.74, *p* < 0.001, *d* = 2.27. Also, participants showed higher *d’* to new faces (*M* = 2.80, *SD* = 0.98) compared to unfair-related faces, *t*(108) = 12.73, *p* < 0.001, *d* = 1.87. However, there was no difference between fair-related and new faces (*p* = 0.24). There was a significant interaction between type of faces and trait anxiety, *F*(2, 206) = 3.99, *p* < 0.03, η_p_^2^ = 0.04. Trait anxiety was negatively correlated with *d’* for fair-related faces (*r* = -2.53, *p* < 0.01), but not with unfair-related faces nor with new faces, *(ps* > 0.46).

**TABLE 2:** Mean values of discrimination indices as a function of different types of faces.

	Hit rates		Misses		Discrimination accuracy (*d’*)		Criterion (C)	
	M	*SD*	M	*SD*	M	*SD*	M	*SD*

**Condition**								
Fair	0.85	0.19	0.15	0.19	2.95	0.86	0.53	0.13
Unfair (Total)	0.67	0.16	0.33	0.16	1.33	0.53	0.25	0.13

	**Correct rejections**		**False alarms**					
New Face	0.90	0.09	0.1	0.09	2.80	0.98	0	0

For response criterion (*C*), there was a significant main effect of the type of faces, *F*(2, 206) = 575.85, *p* < 0.001, η_p_^2^ = 0.85. Paired *t*-tests with Bonferroni correction (2-tailed) revealed that participants made more conservative responses to fair-related (*M* = 0.53, *SD* = 0.13) compared to unfair-related faces (*M* = 0.25, *SD* = 0.13), *t*(108) = 14.40, *p* < 0.001, *d* = 2.15, or compared to new faces (*M* = 0.00, *SD* = 0.00), *t*(108) = 41.47, *p* < 0.001, *d* = 5.77. Also, more conservative responses were made to unfair-related faces compared to new faces, *t*(108) = 20.38, *p* < 0.001, *d* = 2.72. Thus, people not only showed good *d’* but also adopted conservative response criterion to fair-related faces. Also, there was a significant interaction between type of faces and trait anxiety, *F*(2, 206) = 4.55, *p* < 0.02, *η_p_^2^* = 0.04. Trait anxiety was positively correlated with response criterion (*C*) for fair-related faces (*r* = 0.27, *p* < 0.01), but not with unfair-related faces nor with new faces, *(p* > 0.42). Thus, participants with low trait anxiety are more likely to adopt liberal response criterion when recognizing fair-related faces.

## Discussion

The current experiment examined whether fair consideration modulated fair recognition. We utilized the UG task to associate faces with fair and unfair offers and then examined recognition memory for fair-related and unfair-related faces. We also examined the role that trait anxiety played in the recognition of fair-related faces. The current research provides initial evidence that people showed enhanced memory for faces that made fair offers in the economic decision task. However, individuals with high trait anxiety showed reduced memory for fair-related faces compared to those with low anxiety. These results were further confirmed by additional analysis using the signal detection theory. Participants showed higher *d’* and more conservative decision criterion to fair-related faces compared to unfair-related or new faces, and participants with low trait anxiety were associated with higher *d’* and more lax response criterion in response to fair-related faces. The current experiment provided evidence that fair consideration influenced and shaped face recognition, which was modulated by trait anxiety.

The result of the current experiment provided additional evidence to support the ecological theory of face recognition (McArthur and Baron, 1983; Zebrowitz, 2006). The ecological theory of face recognition posits that face recognition plays an important role in guiding interpersonal relationships and navigating the social world (McArthur and Baron, 1983; Zebrowitz, 2006). We predicted that participants would be more likely to remember people who make fair offers in a face recognition task because enhanced recognition of fair-related faces would be highly useful and adaptive in building a social relationship and economic partners for the future. The result of the experiment supported the hypothesis, thereby providing further evidence supporting the ecological theory of face recognition (McArthur and Baron, 1983; Zebrowitz, 2006).

The result of the current experiment is consistent with previous research demonstrating that human memory is easily shaped to conform to the social norm. It has been well established that peoples’ memories are easily altered, distorted, or reconstructed by misinformation, beliefs, moral concerns, and stereotypes (for reviews, see Ross, 1989; Macrae et al., 1994; Roediger, 1996; Hirt et al., 1999; Pizarro et al., 2006; Davis and Loftus, 2007). Among others, justice motivation plays an important role in reconstructing and biasing human memory. People have a long-standing belief—even a need to believe—that the world that they live in is fair and just—termed as *the belief in a just world* (BJW; Lerner and Miller, 1978). BJW can provide a sense of security that the world is a safe place worthy of trust and commitment (Lerner and Miller, 1978). The need to believe in a just and fair world is so ingrained that people are deeply disturbed by evidence that suggests otherwise and make compensatory responses to restore the sense of justice and fairness (Lerner and Miller, 1978). For example, people may characterize victims negatively to be more suitable for a negative consequence that they face (Lerner and Miller, 1978). Furthermore, people reconstructed their memory or selectively remembered the details of the event to be consistent with a BJW (Callan et al., 2009). For example, when participants were asked to recall the amount of a lottery prize for a “bad” or “good” winner, they remembered a smaller lottery prize for the “bad” compared to “good” winner (Callan et al., 2009). Also, when participants were asked to recall an autobiographic memory after experiencing favorable or unfavorable outcomes, they tend to selectively remember events congruent with the outcome. As such, people reconstructed and selectively remembered memory to support BJW. Likewise, the current experiment demonstrated that face recognition is biased to conform to the social norm of fairness. On the other hand, unfair offers create motivational conflict between self-interest and norm enforcements (Feng et al., 2015). Resolving the conflict requires a greater allocation of cognitive resources, which may have depleted resources necessary to make the strong association between faces and unfair offers. As a result, people may have shown reduced memory for unfair faces.

Our results may appear to contradict previous research showing superior memory for untrustworthy faces. For example, in the experiment (Rule et al., 2012), participants were presented with trustworthy and untrustworthy faces in a passive task and then asked to recognize faces presented. Participants demonstrated better memory for untrustworthy compared to trustworthy faces. However, there are several conceptual and methodological differences that may account for the discrepancy between the present experiment and previous research. First, trustworthiness/untrustworthiness and fairness/unfairness are different constructs. They are related, but not identical. Fairness may be one of the attributions that allow someone to be trustworthy. However, trustworthiness may require other attributions, such as honesty and truthfulness. For example, someone can be deceitful and untrustworthy, but fair. In fact, according to the Oxford Dictionary (OED, 2018) fairness is defined as being “the ability to be relied on as honest or trustful.” As such, fairness and trustworthiness are different constructs, which may be operated by different psychological mechanisms; thus they may affect cognitive processes differently. Secondly, there is a substantial methodological difference in which two studies were conducted. Rule et al. (2012) presented facial stimuli in the passive viewing task. However, in the current study, participants learned to associate each face with fairness or unfairness through direct experiences, which may provide more ecological validity. Previous research has shown that the context in which faces are presented plays an important role in face recognition (Buchner et al., 2009). The current experiment provided strong contextual information or source memory by allowing participants to experience direct business dealings with each face, which may have ensured the effect of fairness on face recognition. However, during the passive presentation, participants had to rely on subjective and intuitive judgments to determine the trustworthiness of each face, which may have been influenced by other factors. In fact, concerns were raised regarding the validity and predictive power of people’s judgment on trustworthiness (for review, see Todorov et al., 2008; Stirrat and Perrett, 2010). For example, previous research has shown that people heavily relied on physiognomic features, such as resemblance with emotional expression (Oosterhof and Todorov, 2008) or facial resemblance (DeBruine, 2005) to determine facial trustworthiness. If this is the case, it is possible that the recognition of untrustworthy faces may be confounded by negative emotional expressions; it is well documented that people remember emotionally negative expressions better than emotionally neutral ones (Buchanan, 2007). Furthermore, judgments and recognition on trustworthiness varied depending on the context in which face stimuli were presented (DeBruine, 2005; Brambilla et al., 2018), so did the recognition of untrustworthy faces. Previous research found mixed results of trustworthiness memory when contextual information was provided by verbal description (Mealey et al., 1996; Barclay and Lalumiere, 2006; Rule et al., 2012). Thus, further research is necessary to tease apart the difference between fairness and trustworthiness and underlying cognitive mechanisms.

The current research provided evidence that individual differences in anxiety modulated the accuracy of fair-related memory, such that individuals with high trait anxiety showed reduced memory for fair-related faces compared to those with low trait anxiety. Previous research reported that clinically anxious participants accepted more unfair offers than healthy controls (Grecucci et al., 2013). Fairness was considered socially preferable and normative (Feng et al., 2015). Thus, when the social norm of fairness is violated, people are willing to punish fairness norm violators at the cost of losing money in the UG task. However, individuals with high anxiety want to avoid social confrontation, thus being more willing to accept unfair offers (Grecucci et al., 2013). To avoid anxiety associated with confronting and punishing fair norm violators, individuals with high anxiety might need to minimize the advantage of fairness and be less mindful of the violation of the fairness norm. As a result, individuals with high anxiety may show reduced memory for the proposers who made fair offers. Also, due to poor memory of fair-related stimuli, individuals with high anxiety are less likely to take advantage of fair business dealings and trades in the future and to form a meaningful social relationship with moral and ethical individuals.

The correlation between low trait anxiety and more lax response criterion may suggest that participants with low trait anxiety may experience greater familiarity of fair-related faces. According to dual-processing theories of recognition memory, two processes—recollection and familiarity—are involved in memory performance (see Yonelinas, 2002. for a review). Recollection refers to the recollection evoked by an old item that was specifically presented before, whereas familiarity refers to a sense of familiarity that people experience to an item without specific recollection of previous encounter (Knowlton and Squire, 1995). Yonelinas (2002) demonstrated that changes in response criterion affect familiarity, but not recollection. Thus, the fact that individuals with low trait anxiety adopted more lax response criterion suggests that individuals with low trait anxiety were more likely to use familiarity-based processes when responding to fair-related faces. However, more reliance on familiarity-based processing did not compromise their ability to accurately recognize the fair-related faces as individuals with low trait anxiety demonstrated high *d’*. Nevertheless, we did not directly employ the retrieval procedures to differentiate the recollection- and familiarity-related processes. Thus, the caution should be made to interpret the relationship between anxiety and response criteria using the recollection-familiarity frameworks.

It should be noted that we did not find that anxiety modulated the acceptance rate of unfair offers during the UG task. Previous research was conducted on patients who have clinically diagnosed anxiety disorders (Grecucci et al., 2013). However, the current research was conducted using healthy individuals with high trait anxiety scores. In fact, previous research which studied participants with subclinical anxiety did not find that anxiety modulated acceptance rates (Luo et al., 2014). Thus, it is possible that the severity of anxiety plays an important role in economic decision making.

Neuroimaging evidence (Tabibnia et al., 2008) has shown that fair offers in the UG task elicited greater activation in the ventromedial prefrontal cortex (vmPFC), a core region associated with integrating values into decision-making (Hare et al., 2010; Hunt et al., 2012). Fair offers elicited the continuous activation of the vmPFC, which remained strong after controlling for the monetary payoff, suggesting that the vmPFC is involved in processing positive norm values associated with fair offers (Tabibnia et al., 2008; Roy et al., 2012). Furthermore, some neuroimaging studies have reported that the vmPFC is implicated in memory consolidation (for reviews, see Nieuwenhuis and Takashima, 2011). Thus, the vmPFC is involved in both assessing positive norm values associated with fair offers and memory consolidation of information, which may provide neurological mechanisms of memory bias favoring fair offers in the UG task. However, neuroimaging evidence will be necessary to clarify further the neural mechanisms underlying the relationship between fair consideration and human memory.

## Conclusion

The current research suggests that face recognition is modulated by fair consideration—a social norm that people inherently prefer to confirm. People showed enhanced memory for faces that made fair offers, which was further confirmed by the signal detection theory. Furthermore, we provided evidence that high trait anxiety was associated with reduced memory for fair-related faces, which might place individuals with high trait anxiety to be disadvantageous in social and economic situations.

## Ethics Statement

This study was carried out in accordance with the recommendations of ‘Azusa Pacific University Institutional Review Board (IRB)’ with written informed consent from all subjects. All subjects gave written informed consent in accordance with the Declaration of Helsinki. The protocol was approved by the ‘Azusa Pacific University Institutional Review Board (IRB).’

## Author Contributions

GP designed the experiments and collected data with EJ. GP analyzed the data and wrote the manuscript with critical edits from BM and EJ.

## Conflict of Interest Statement

The authors declare that the research was conducted in the absence of any commercial or financial relationships that could be construed as a potential conflict of interest.

## Footnotes

1 The behavioral and cardiovascular data from one participant was lost due to equipment failure and a computer error. According to *G*Power* (Faul et al., 2007), the sample size of 105 was proposed to detect medium effects of about *p* = 0.25. The data are available online at:https://osf.io/5ch4n/

2 We conducted preliminary analyses to explore whether these personality questionnaires modulated the acceptance rates and face recognition. No significant result was obtained between scores from these personality questionnaires and face recognition (*ps* > 0.33). However, there was a marginally significant interaction between the acceptance rates and the Duke University Religion Index, *F*(3, 321) = 2.52, *p* = 0.06. We plan to examine the relationship in future studies.

3 We also conducted a 4 (Offers: $5-$5, $7-$3, $8-$2, $9-$1) **×** 2 (Gender: men, women) mixed factorial analysis of variance (ANOVA) to examine the gender effect. No interaction was observed (*p* = 0.34).

4 We conducted an analysis on Reaction Times (RT) that excluded incorrect trials. The trimmed RTs were subjected to 3 (Type of Faces: fair-related, unfair-related, new faces) repeated measures analysis of covariance (ANCOVA) with z-standardized trait anxiety (STAI-trait), which yielded no significant interaction (*p* = 0.11). Additional analysis found no gender effect, (*p* = 0.97).

5 Following the recommendation of Snodgrass and Corwin (1988), we used *d’* and *C* for recognition memory {*d’* = [*z*(Hit)−*z*(FA)], *C* = −[*z*(Hit)+*z*(FA)]/2}.
